# Impact of age, sex, and joint form on degenerative lesions of the sacroiliac joints on CT in the normal population

**DOI:** 10.1038/s41598-021-85303-5

**Published:** 2021-03-15

**Authors:** Katharina Ziegeler, Virginie Kreutzinger, Torsten Diekhoff, Robert Roehle, Denis Poddubnyy, Matthias Pumberger, Bernd Hamm, Kay Geert A. Hermann

**Affiliations:** 1 Department of Radiology, Charité-Universitätsmedizin Berlin, Berlin, Germany; 2 Department of Radiology, Vivantes Klinikum am Friedrichshain, Berlin, Germany; 3 Institute of Biometry and Clinical Epidemiology, Charité-Universitätsmedizin Berlin, Berlin, Germany; 4 Division of Gastroenterology, Infectious Diseases and Rheumatology, Charité-Universitätsmedizin Berlin, Berlin, Germany; 5 Center for Musculoskeletal Surgery, Charité-Universitätsmedizin Berlin, Berlin, Germany

**Keywords:** Musculoskeletal system, Musculoskeletal system, Osteoarthritis

## Abstract

Degeneration of the sacroiliac joints (SIJs) is a common finding, while its underlying cause and development remain incompletely understood. The aim of this investigation was to describe the spatial distribution of degenerative SIJ changes across age groups and to investigate for the first time their relationship to anatomical form and sex. For this IRB-approved investigation, demographic data of 818 patients without SIJ disease were retrieved from electronic patient records. High-resolution computed tomography (CT) datasets of all patients were analysed retrospectively for seven predefined age groups (ten-year increments, from < 25 to ≥ 75). A structured scoring system was applied to assess sclerosis, osteophytes, joint space alterations, and anatomical form. Chi-square tests were used to compare frequencies of degenerative lesions, and logistic regression analyses were performed to investigate associations between demographic data, anatomical form, and the presence of structural lesions. Sclerosis and osteophytes were common findings, with an overall prevalence of 45.7% and 46.8%, respectively. Female sex had an odds ratio (OR) of 0.15 (95% CI: 0.08–0.27) for the presence of ventral osteophytes and of 4.42 (95% CI: 2.77–7.04) for dorsal osteophytes. Atypical joint forms were significantly more prevalent in women with 62.1% vs. 14.1% in men (p < 0.001). Accessory joints increased the likelihood of dorsal sclerosis (OR 2.735; 95% CI 1.376–5.436) while a typical joint form decreased its likelihood (OR 0.174; 95% CI 0.104–0.293). Sex and anatomical joint form have a major impact on the development of degenerative lesions of the SIJs and their spatial distribution.

## Introduction

Low back pain is a very common condition affecting up to 80% of adults at least once during their lifetime^1,2^. In up to 30% of these patients, the sacroiliac joint (SIJ) may play a role in the course of the disease^3,4^. Degeneration of the SIJ as detected by computed tomography (CT), however, is a common finding even in populations with few or no symptoms^5^. As structural joint changes are commonly considered surrogates for the presence or progression of both mechanical joint disease^6^ and axial spondyloarthritis^7,8^, robust data on their prevalence in the normal population are essential for correct diagnosis and classification. A number of studies investigated such degenerative changes in asymptomatic individuals, both in and ex vivo^5,9–13^: overall, these studies showed the prevalence of sclerosis and osteophytes to increase and the width of the joint space to decrease with age. There are, however, a number of cofactors that contribute to mechanical joint stress, which have thus far received less attention in imaging research, among them sex differences and anatomical variants. It has long been established that the pelvic skeleton exhibits significant sexual dimorphism^14^: the male pelvis is typically longer and narrower with a more conical shape than the female pelvis. Additionally, the female sacrum is typically wider with a less smooth surface^15^, and the ligamentous stabilization of the joint loosens under the influence of relaxin during pregnancy^16^.

Apart from sex differences in the pelvic skeleton, there are five distinct anatomical variants of the SIJ that have been described in the literature^17^: the accessory SIJ^18^, the sacroiliac complex, the bipartite iliac bone plate, semicircular defects, and ossification centres in the sacral wings. Although there are case reports of symptomatic cases of accessory SIJs, the full spectrum of anatomical variants has thus far not been evaluated for their significance in biomechanical joint stress.

Previous studies were conducted more than twenty years ago, used soft CT reconstruction kernels not intended for bone imaging with large slice thickness, only analyzed axially formatted images, or examined very small numbers of patients. To the best of our knowledge, no investigation has so far demonstrated a relationship of anatomical form and patient-specific factors with the spatial distribution of degenerative lesions, although we may plausibly hypothesize that different joint forms lead to differences in biomechanical load.

The aim of this study was to describe degenerative lesions of the sacroiliac joints across age groups in a population without sacroiliac joint disease and to investigate their relationship with anatomical form, sex, and other patient-specific factors.

## Materials and methods

### Sample size estimation and inclusion and exclusion criteria

**Figure 1:**
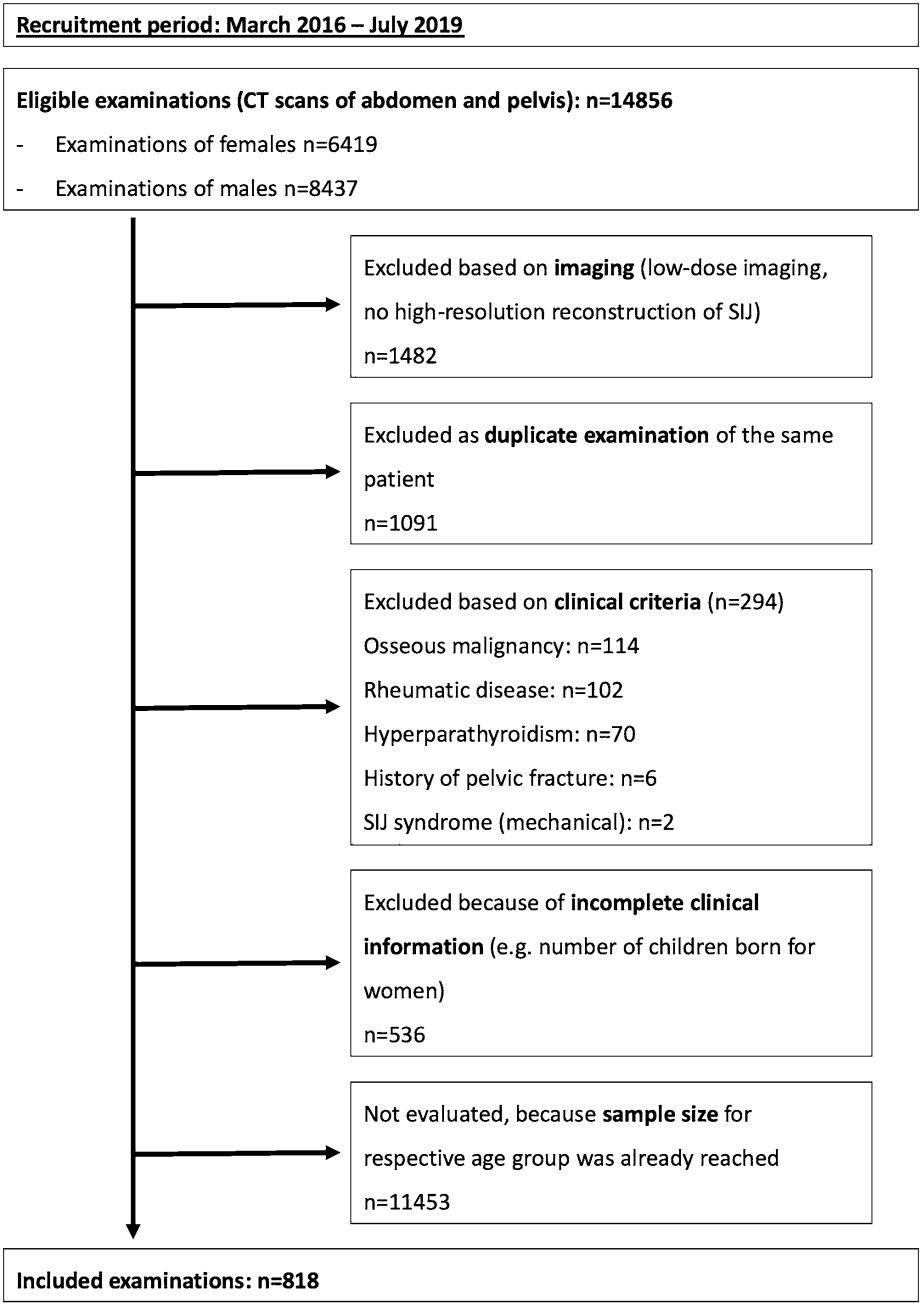
Flow diagram of patient enrollment.

This study was approved by the ethics review board of the Charité-Universitätsmedizin Berlin (EA1/300/19) and conducted in accordance with the Declaration of Helsinki as well as local legislation and ethical standards; due to the retrospective nature of the investigation, the ethics review board waived individual written informed consent. Before we started this retrospective analysis, a sample size estimation was performed by the Institute of Biometry and Clinical Epidemiology using dedicated software (nQuery Version 7.0, StatSols, Ohio, USA). Assuming a conservative estimate of a lesion frequency of 3% with an alpha-error of 5% and a power of 80%, a sample size of 815 was calculated as sufficient to ascertain that the lesion rate of erosions is below 5% using a one-sample Chi^2^-test. Results for this endpoint (erosions) are the subject of second analysis regarding inflammatory lesions, which is to be published separately. The analyses shown here investigate further, exploratory questions. Eligible for enrolment in this study were patients who underwent a computed tomography (CT) scan of the pelvic region from March 2016 through July 2019 in our department of radiology. It was planned to include male and female patients in equal proportions in seven predefined age groups in ten-year increments from < 25 to ≥ 75 years. Inclusion was performed per age group and sex separately—CT datasets were evaluated consecutively based on scan date and included or excluded according to predefined criteria until the required sample size was reached. A flow diagram of patient enrolment is given in Fig. 1. Patients were excluded based on imaging if a low-dose CT technique was used or the datasets had missing or incomplete high-resolution secondary reconstructions of the SIJs. Furthermore, all patients with known SIJ disease/SIJ syndrome, known back pain, status post surgery of the lumbar spine, known rheumatic disease, hyperparathyroidism, bone malignancy (both primary and metastatic), or pelvic fractures were excluded, as were patients with missing electronic records (e.g., outpatients) or lacking information on prior diagnoses or number of children born for women.

### Patient-specific factors

Electronic patient records were searched for the parameters listed in the preceding section as well as nutritional status (slim, normal, obese), inflammatory bowel disease, cutaneous psoriasis, history of uveitis, and indication for the CT examination (oncological staging, search for infectious focus, trauma, bleeding, other). All CT and demographic patient data were anonymized using dedicated software in order to rule out identification of individual patients.

### Imaging technique

During the recruitment period, all pelvic CT examinations routinely included a secondary reconstruction of the pelvic skeleton—the raw data were reconstructed in a sharp bone kernel with 0.5 mm slice thickness as an isometric volume. All CT datasets were scored by one radiologist with 5 years of experience in musculoskeletal radiology (junior reader) who was blinded to all clinical data. Images were read in random order using dedicated software (Horos v3.3.6, The Horos Project, public license) with dynamic multiplanar reconstruction capacity. Volume image datasets were scored predominantly in an oblique coronal plane (parallel to the long axis of the second sacral vertebra) and with bone window settings, but the readers were permitted to freely adjust the reconstruction plane, window level and width as well as magnification.

### Scoring system

**Figure 2:**
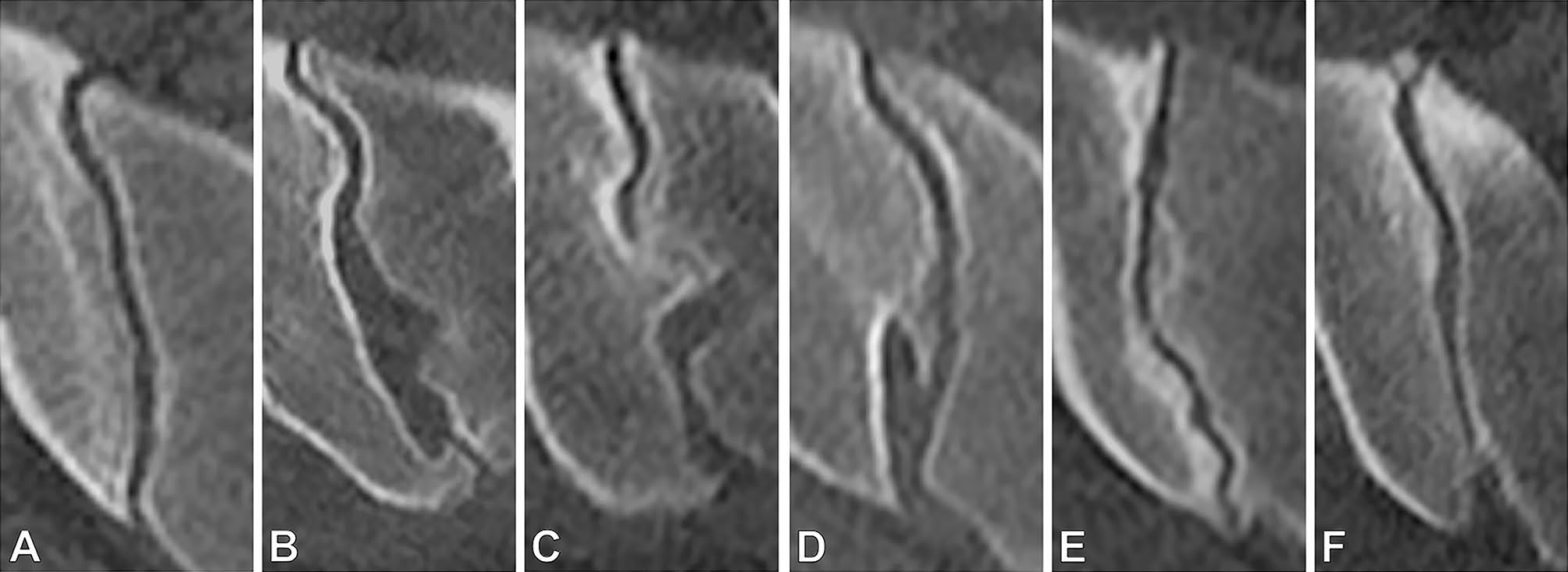
Imaging example from the atlas: anatomical variants. Right sacroiliac joint in axially reconstructed volume. **(A)** Typical joint form. **(B)** Accessory joint facet dorsally. **(C)** Convex iliac notch with corresponding sacral grooving, forming the iliosacral complex. **(D)** Bipartite iliac bone plate. **(E)** Crescent-shaped ilium with corresponding convexity of the sacrum. **(F)** Sacral ossification center in the ventral aspect of the joint.

Prior to analysis of CT datasets, a scoring system was compiled, expanding on previously published work^19^. In short, the sacroiliac joint complex was divided into 24 regions (12 on each side) and, for each region, the presence of sclerosis was recorded (1 = possible/very little sclerosis; 2 = marked sclerosis). Additionally, joint space alterations (per side; 1 = possible widening/narrowing; 2 = pseudowidening/narrowing; 3 = partial ankylosis; 4 = complete ankylosis) and osteophytes (ventral and dorsal, separately for each side; 1 = small osteophyte (< 5 mm); 2 = larger osteophyte (> 5 mm); 3 = bridging osteophyte) were scored. Lastly, the anatomical form of each joint was recorded, based on a classification proposed by Prassopoulos et al.^17^, who described six distinct anatomical variants: accessory joint, iliosacral complex, bipartite bony plate of the ilium, crescent-shaped iliac bone, semicircular defects, and ossification centers of the sacral wings. Before readers assessed the study patients, an atlas of exemplary cases not included in this analysis was compiled, access to which was permitted during image reading; an excerpt from the atlas is presented in Fig. 2. Scoring results were recorded in a RedCap (Vanderbuilt University, Nashville, USA) database. In addition, the reader underwent a teaching session including 15 test cases (not included in the study) with a consultant radiologist with expertise in MSK radiology (senior reader). Both junior and senior reader also scored a random sample of 50 study patients (in case of the junior reader a second time) to calculate inter- and intrareader reliability.

### Statistical analysis

Statistical analysis was carried out using SPSS version 24 (IBM Corporation, New York, USA). Scoring results were summarized separately as sum scores for each structural lesion on the patient level. On the patient level, positivity for sclerosis was defined as a sum score (sclerosis) > 2, positivity for osteophytes was defined as a sum score (osteophytes) > 1, and positivity for joint space alterations was defined as a sum score (joint space) ≥ 1. Frequencies of structural lesions and anatomical variants were compared between subgroups using Chi-square tests. For presence of sclerosis, joint space alterations, and osteophytes (separately for ventral and dorsal aspects of joints), logistic regression analyses were performed. Covariables were sex, age, weight category, and anatomical form in all patients and number of children born in women as relaxin-induced ligamentous SIJ instability of the SIJs might contribute to mechanical joint disease in women^16,20^ as well as inflammatory bowel disease and cutaneous psoriasis because of their association with sacroiliitis^21^.

Intraclass correlation coefficients (ICCs) were calculated for interreader reliability using a two-way mixed model ICC(3,2)^22^ on sum scores for each lesion type and on overall anatomical form; the same model was used to assess intrareader reliability. A significance level of p < 0.05 was assumed for all tests.

## Results

### Patients and clinical findings

Approximately half of the CT examinations in the study population were performed for oncological staging (389/818; 47.6%) while a third was performed for identification of an infectious focus (299/818; 36.6%); 27 patients (3.3% of 818) were examined for bleeding or abdominal trauma and 103 (12.6% of 818) for other indications, e.g., vascular occlusion or preoperative planning before abdominal surgery. As per study design, male and female patients were included in equal proportions in each age group. Clinical characteristics of the study patients are summarized in Table 1. More than half of the patients aged 45 years and older were classified as overweight (55.3%; 273/494). More than half of the women (245/401) had given birth to at least one child, 16.2% (65/401) had given birth to three or more children. Both inflammatory bowel disease and cutaneous psoriasis were very rare, occurring in 3.5% (29/818) and 0.9% (7/818) of our patients, respectively. There were no patients with a documented history of uveitis.

**Table 1:** Patient characteristics by age group. For parity, percentages are given for women of the respective age group only, not for entire age group.

Age group	n	Sex		Overweight	Parity (female population only)	
		Male	Female			
					1–2	≥ 3
< 25	77	58.4% (45/77)	41.6% (32/77)	11.7% (9/77)	3.1% (1/32)	3.1% (1/32)
25–34	126	50.0% (63/126)	50.0% (63/126)	21.4% (27/126)	12.7% (8/63)	3.2% (2/63)
35–44	121	49.6% (60/121)	50.4% (61/121)	26.4% (32/121)	50.8% (31/61)	18.0% (11/61)
45–54	123	50.4% (62/123)	49.6% (61/123)	52.0% (64/123)	47.5% (29/61)	19.7% (12/61)
55–64	125	49.6% (62/125)	50.4% (63/125)	55.2% (69/125)	60.3% (38/63)	22.2% (14/63)
65–74	122	51.6% (63/122)	48.4% (59/122)	55.7% (68/122)	59.3% (35/59)	25.4% (15/59)
≥ 75	124	50.0% (62/124)	50.0% (62/125)	58.1% (72/124)	61.3% (38/62)	16.1% (10/62)
Total	818	51.0% (417/818)	49.0% (401/818)	41.7% (341/818)	44.9% (180/401)	16.2% (65/401)

### Frequency of structural lesions and variant joint form

The frequency of degenerative lesions (as defined in the Methods section) per age group is given in Table 2. Sclerosis was most frequent in the age group ≥ 75 years, exhibiting a steady increase in prevalence with age with a small second peak in the age group 35–44 years; overall, 374 patients (of 818; 45.7%) exhibited sclerosis.

**Table 2:** Frequency of degenerative lesions by age group. Frequencies are given relative to the respective age group with absolute numbers in brackets.

Age group	Sclerosis	Osteophytes	Joint space alterations
< 25	16.9% (13/77)	10.4% (8/77)	1.3% (1/77)
25–34	35.7% (45/126)	15.1% (19/126)	0.0% (0/126)
35–44	52.1% (63/121)	33.1% (40/121)	5.0% (6/121)
45–54	43.1% (53/123)	52.8% (65/123)	8.1% (10/123)
55–64	46.4% (58/125)	54.4% (68/125)	8.8% (11/125)
65–74	50.8% (62/122)	71.3% (87/122)	12.3% (15/122)
≥ 75	64.5% (80/124)	77.4% (96/124)	26.6% (33/124)
Total	45.7% (374/818)	46.8% (383/818)	9.3% (76/818)

As with sclerosis, the frequency of osteophytes increased in a nearly linear fashion, peaking at 77.7% (96/124) in the age group ≥ 75 years with an overall frequency of 46.8% (383/818) in the entire population. Joint space alterations were very rare in younger patients, with only one case being younger than 35 years, but increased in frequency with age up to 26.6% (33/124) in patients ≥ 75 years. Altogether, 9.3% (76/818) of patients exhibited joint space alterations. Only one patient had complete ankylosis of one sacroiliac joint. Anatomical variants were significantly more prevalent in women, of whom only 37.9% (152/401) had a typical joint form vs. 85.9% (358/417) of men (p < 0.001). The most common anatomical variant was a bipartite iliac bone plate (11.1%, 91/818; 88 women, 3 men), followed by an accessory joint (8.3%, 68/818; 51 women, 17 men). All other joint forms were rare (less than 20/818) in our cohort. The results are summarized in Table 3.

**Table 3:** Frequencies of joint forms. P-values derived from Chi^2^ tests.

Joint form	Males	Females	p
Typical joint form	85.9% (358/417)	37.9% (152/401)	< 0.001
Accessory joint	4.1% (17/417)	12.7% (51/401)	< 0.001
Iliosacral complex	1.2% (5/417)	4.5% (14/401)	0.036
Bipartite ilium	0.7% (3/417)	21.9% (88/401)	< 0.001
Crescent-shaped ilium	0.7% (3/417)	3.0% (12/401)	0.018
Semicircular defects	0.2% (1417)	0.2% (1/401)	> 0.999
Sacral ossification centers	0.5% (2/417)	0.5% (2/401)	> 0.999
Other joint form	6.7% (28/417)	20.2% (81/401)	< 0.001

### Localization of degenerative lesions

**Figure 3:**
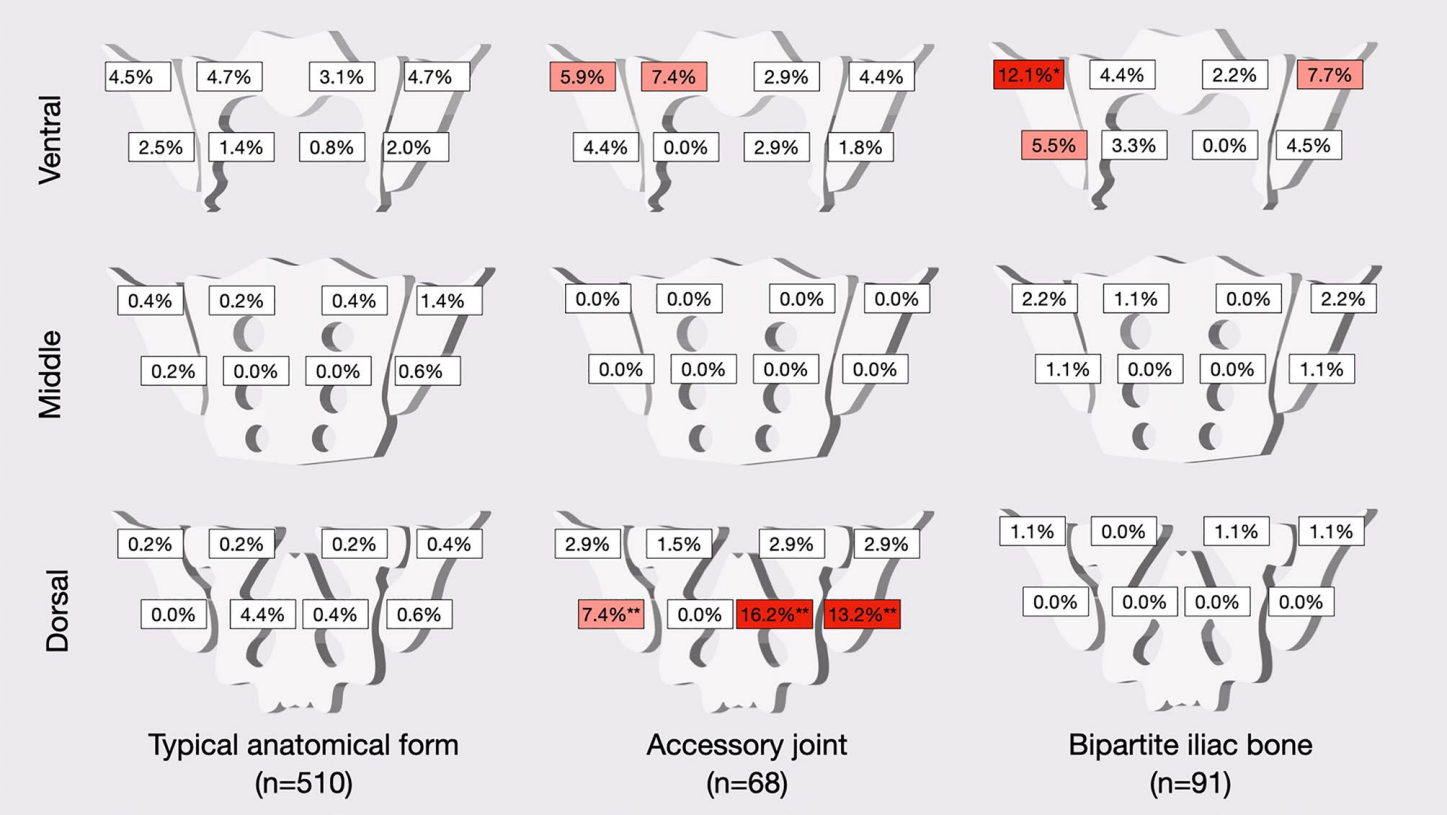
Frequency of sclerosis per region for different anatomical forms (%)*.* Upper row: ventral portion of the joint (regions 1–8). Middle row: middle portion of the joint (regions 9–16). Lower row: dorsal portion of the joint (regions 17–24). Columns represent anatomical types. Frequencies smaller than 5% are given in white boxes, frequencies of 5 to 10% are given in light red, frequencies above 10% are given in dark red boxes. Significantly higher frequencies (compared to both other groups using Chi-squared tests) are marked with asterisks: *p < 0.05/**p < 0.001.

Localization of degenerative lesions was investigated separately for different anatomical variants, specifically for the typical anatomical form, the accessory joint, and the bipartite iliac joint plate, as other variants were very rare. A graphical summary of the frequency of sclerosis in patients with different joint forms is given in Fig. 3.

**Figure 4:**
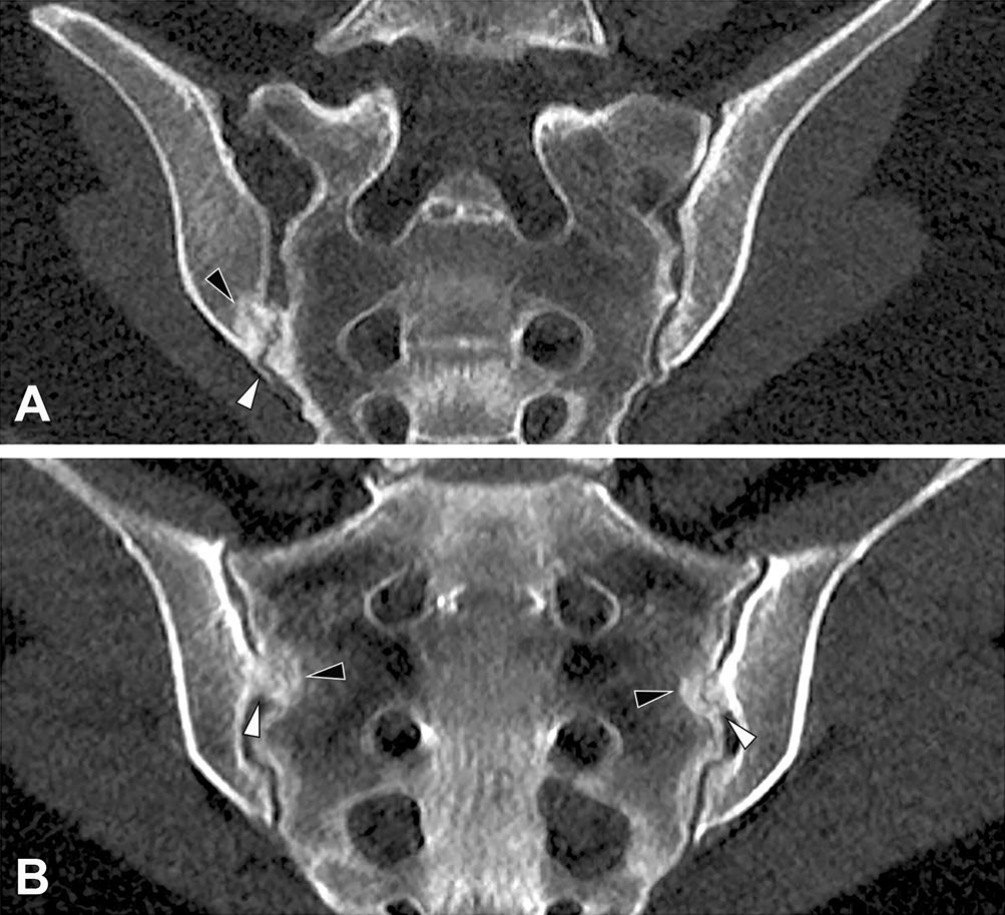
Imaging examples. **(A)** Female patient with bilateral accessory joints, paracoronal reconstruction. Note the accessory joint facet (white arrowhead) with associated periarticular sclerosis (black arrowhead). **(B)** Female patient with bilateral iliosacral complex, paracoronal reconstruction. Note the convex ilium notches (white arrowheads), again with associated periarticular sclerosis (black arrowheads).

In patients with typical joint anatomy, sclerosis did not show a significant predilection for any region with an overall low occurrence of < 5% in all regions. In patients with an accessory joint, sclerosis was most pronounced in the dorsal and caudal portions of both ilium and sacrum—these differences were statistically significant. Patients with a bipartite iliac bone plate exhibited sclerosis mainly in the ventral cranial part of the ilium and on the right side, their frequency was significantly higher (11/91; p = 0.005). Distribution of osteophytes also differed between patients with different joint forms. Ventral osteophytes were seen in 30.2% (154/510) of patients with a typical joint form and in 35.3% (24/68) and 14.3% (13/91) of patients with accessory joints and bipartite bone plate respectively—the difference in frequency was statistically significant (p = 0.048). Dorsal osteophytes were significantly (p < 0.001) more common in patients with accessory joints (58.8%; 40/68) and bipartite iliac bone plate (53.8%; 49/91) than in those with a typical joint form (27.8%; 142/510). Joint space alterations were seen in 9.6% (49/510) of patients with typical joint form, 16.2% (11/68) of patients with an accessory joint and 4.4% (4/91) of patients with a bipartite bone plate. Imaging examples from the study cohort are provided in Fig. 4.

### Multivariable regression analysis

Results of multivariable logistic regression analysis are given in Table 4.

**Table 4:** Regression analysis.

		Sclerosis (ventral) [OR, 95% CI]	Sclerosis (middle) [OR, 95% CI]	Sclerosis (dorsal) [OR, 95% CI]	Osteophytes (ventral) [OR, 95% CI]	Osteophytes (dorsal) [OR, 95% CI]	Joint space alterations [OR, 95% CI]
Covariates	Sex [female]	1.1 (0.7–1.5)	1.0 (0.7–1.5)	1.2 (0.7–1.2)	**0.2 (0.1–0.3)**	**3.7 (2.4–5.7)**	0.6 (0.3–1.1)
	Parity	1.1 (1.0–1.3)	1.0 (0.9–1.1)	1.0 (0.8–1.1)	0.9 (0.8–1.1)	1.0 (0.9–1.2)	0.9 (0.7–1.3)
	Age [decades]	**1.1 (1.0–1.2)**	**1.1 (1.1–1.3)**	**1.1 (1.0–1.2)**	**2.0 (1.7–2.2)**	**1.4 (1.3–1.6)**	**1.8 (1.5–2.1)**
	Weight [cat.]	**1.5 (1.1–1.9)**	1.2 (1.0–1.6)	**1.5 (1.1–2.0)**	1.2 (0.9–1.6)	**1.5 (1.1–2.0)**	0.7 (0.5–1.2)
	IBD	0.7 (0.4–1.1)	0.9 (0.6–1.5)	1.3 (0.7–2.2)	1.1 (0.4–3.4)	1.3 (0.5–3.3)	n.a.
	Pso	1.2 (0.3–5.8)	4.5 (0.9–24.1)	1.4 (0.2–8.9)	4.3 (0.4–48.8)	2.2 (0.5–10.3)	2.1 (0.3–13.1)
	Typ J	1.2 (0.8–2.0)	0.7 (0.5–1.2)	**0.2 (0.1–0.3)**	0.5 (0.3–0.9)	0.6* (0.4–1.0)	1.1 (0.5–2.7)
	Acc J	1.2 (0.6–2.3)	0.5 (0.3–1.0)	**2.7 (1.4–5.4)**	0.7 (0.3–1.6)	0.9 (0.5–1.8)	1.5 (0.5–4.3)
	ISC	0.5 (0.2–1.4)	1.1 (0.4–3.1)	**0.2 (0.0–0.7)**	3.6* (1.0–12.3)	0.7 (0.2–2.2)	1.8 (0.3–10.8)
	Bip Ilium	1.1 (0.6–2.0)	1.3 (0.7–2.3)	0.6 (0.3–1.1)	0.6 (0.3–1.4)	0.9 (0.5–1.6)	0.7 (0.2–2.7)
	Cresc J	1.7 (0.6–5.4)	1.7 (0.6–5.2)	0.3 (0.1–1.1)	3.6 (0.9–14.4)	0.7 (0.2–2.5)	1.4 (0.1–14.0)
	Sem Def	0.9 (0.1–15.0)	n.a.	n.a.	1.7 (0.1–33.7)	0.9 (0.0–19.6)	n.a.
	Oss Sac	6.2 (0.6–63.7)	0.9 (0.1–8.8)	n.a	2.4 (0.1–46.2)	n.a	n.a
	Other	0.7 (0.2–2.3)	0.5 (0.2–1.9)	0.4 (0.1–1.2)	0.6 (0.1–2.5)	1.8 (0.5–6.0)	1.8 (0.3–10.2)

Results of binary logistic regression analyses.

Significant ORs with 95% CIs excluding 1.0 are written in bold.

*OR* odds ratio, *CI* confidence interval, *IBD* inflammatory bowel disease, *Pso* cutaneous psoriasis, *Typ J* typical joint form, *Acc J* accessory joint, *ISC* iliosacral complex, *Bip Ilium* bipartite ilium, *Cresc J* crescent-shaped ilium, S*em Def* semicircular defects, *Oss Sac* sacral ossification centers, *other* other joint form.

Three different models were calculated for sclerosis of the ventral, middle, and dorsal SIJ, yielding a model accuracy, expressed as Nagelkerke’s R^2^, between 0.055 (middle) and 0.259 (dorsal) sclerosis. This means that 5.5% to 25.9% of the variation in sclerosis are explained by changes in the examined covariates. Analysis showed age (in decades) to be positively associated with sclerosis in the middle and ventral portions of the SIJ with odds ratios (OR) of 1.149 (95% CI 1.055–1.251; p = 0.001) and 1.093 (95% CI 1.009–1.184; p = 0.029), respectively. Weight was positively associated with ventral and dorsal sclerosis with ORs of 1.475 (95% CI 1.148–1.894; p = 0.002) and 1.484 (95% CI 1.077–2.045; p = 0.016). In terms of joint form, we found a typical joint form to be negatively associated with dorsal sclerosis (OR 0.174; 95% CI 0.104–0.293; p < 0.001) and an accessory joint to be a strong positive cofactor for the same finding (OR 2.735; 95% CI 1.376–5.436; p = 0.004). Neither sex nor parity were found to have a significant impact on sclerosis.

**Figure 5:**
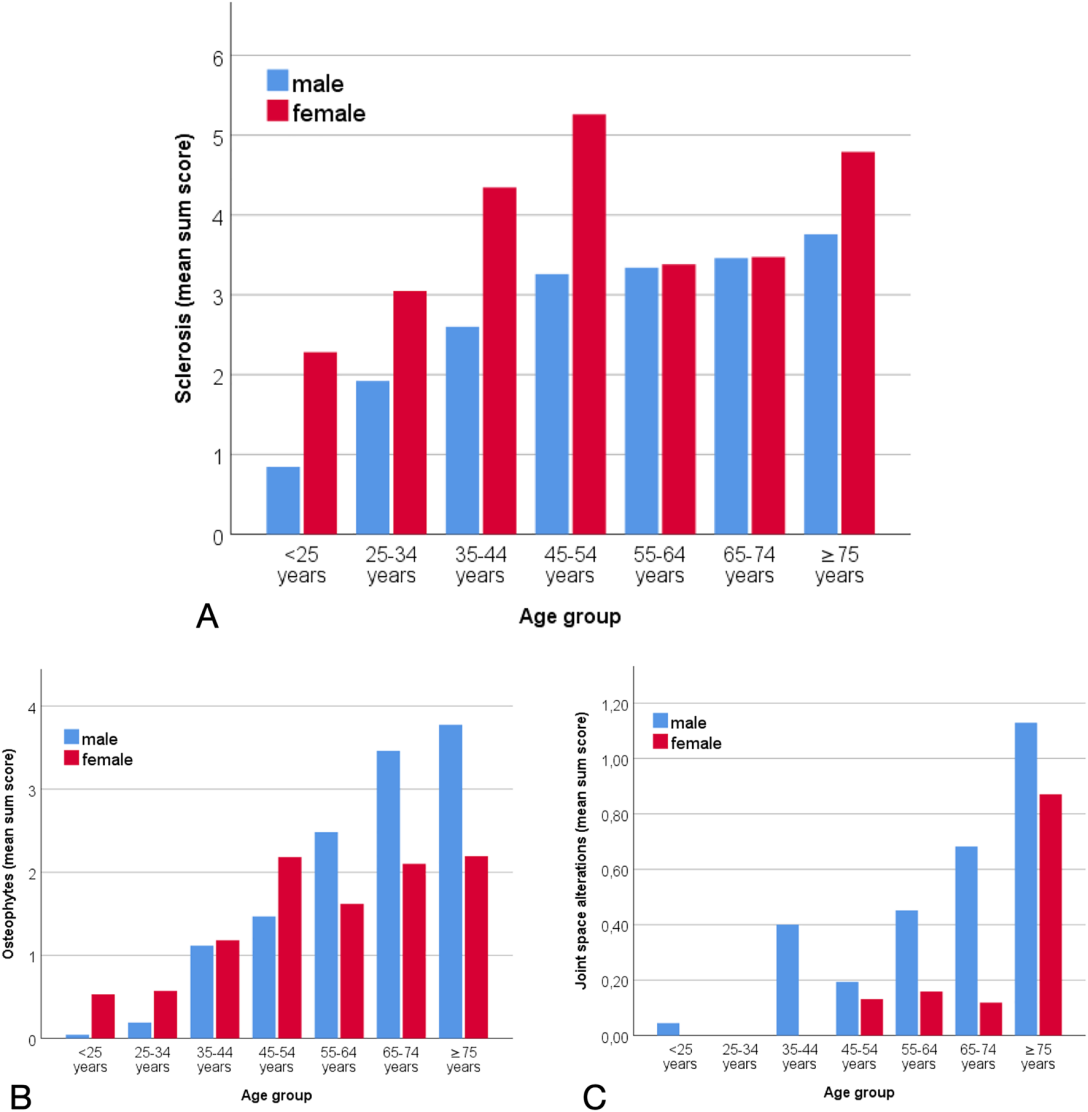
Extent of structural lesions (mean sum score) per age group. Maximum sum score = 48. **(A)** Sclerosis. **(B)** Osteophytes. **(C)** Joint space alterations.

For joint space alterations (Nagelkerke’s R^2^ = 0.192), the only significant association was found for age (in decades) with an OR of 1.768 (95% CI 1.491–2.096; p < 0.001). The model for ventral osteophytes produced a Nagelkerke’s R^2^ of 0.402; significant covariates were female sex (OR 0.164, 95% CI 0.095–0.282; p < 0.001), age in decades (OR 1.974, 95% CI 1.748–2.229; p < 0.001), and an iliosacral complex (OR 3.558, 95% CI 1.033–12.258; p = 0.044). For dorsal osteophytes (Nagelkerke’s R^2^ = 0.285) significant covariates were sex (OR 3.703, 95%CI 2.427–5.650; p < 0.001), age in decades (OR 1.409, 95%CI 1.280–1.550; p < 0.001) and weight category (OR 1.511, 95%CI 1.137–2.008; p = 0.004). Results for the extent of sclerosis, osteophytes and joint space alterations (mean sum scores) in men and women by age group are displayed in Fig. 5. The figure shows a steady increase in sclerosis with age in males and a peak in women aged 45–54 years.

### Inter- and intrareader agreement

In order to test for robustness of our scoring system, both inter- and intrareader reliability were computed using ICCs. Interreader reliability was good^22^ with a mean ICC of 0.778 (range: 0.574–0.904; p < 0.001), and intrareader reliability was also good with a mean ICC of 0.834 (range: 0.766–0.876; p < 0.001).

## Discussion

To our knowledge, this is the first study to systematically explore the association of anatomical variants, patient-specific factors, and degenerative lesions of the SIJs as detected by dedicated computed tomography in a large population of patients without SIJ-related symptoms. We found that specific anatomical configurations have a significant impact on the development of degenerative SIJ lesions and that sex plays a major role in the spatial distribution of these lesions.

Overall, prevalence of sclerosis showed an almost linear increase with age. These findings are in line with the results by Eno et al.^5^, Faflia et al.^10^ and Shibata et al.^11^, who also found linear increases of frequency and extent of degenerative lesions, applying different systems of assessment. In terms of extent of sclerosis, we found a peak in females aged 45 to 54 years that was not mirrored in males, who displayed a steadier increase with age. This finding is especially significant as sclerosis is considered a structural lesion in axial spondyloarthritis, a disease which is often first diagnosed in this particular age group^23^.

The relative frequencies of atypical joint variants in our population differ somewhat from those reported by Prassopoulos et al. in 534 patients^17^, who found a bipartite iliac bony plate in only 4.1% (vs. out 11.1%) of their patients, indicating a high variance in the frequency of this finding. However, our data confirm a difference in sex distribution of this joint form.

The spatial distribution of SIJ degeneration differs substantially between men and women: female sex was associated with only a fifth of the risk of male sex to exhibit ventral osteophytes (OR 0.2) but with more than a fourfold risk to exhibit dorsal osteophytes (OR 4.4) This may be attributable to the sex- specific pelvic angle of aperture and higher joint flexibility in women^24^ and was found to be independent of the number of children born. Peripartum changes of the sacroiliac joints have thus far been mainly described in MR imaging^25,26^, although Faflia et al. described a trend toward more extensive joint space alterations in pelvic CT scans of overweight, multiparous women^10^. In terms of anatomical variants, an iliosacral complex increased the likelihood of developing ventral osteophytes, while a typical joint form decreased the likelihood of exhibiting sclerosis and dorsal osteophytes. Additionally, sclerosis was significantly more prevalent in the dorsal and caudal SIJ portions in patients with an accessory joint, which is a plausible finding as an additional articulation with potentially atypical biomechanical loading may lead to sclerosis.

Both inter- and intrareader agreement were good, which suggests that the 24-region model, first proposed by Diekhoff et al.^19,27^, is a practical and robust approach for capturing the complex anatomy of the SIJ in semiquantitative surveys of joint alterations.

Despite careful planning and execution, our study has some limitations. As only patients with complete information in their electronic health records were included, some selection bias may have been present. Based on its low prevalence during patient enrolment there is reason to suspect that low back pain and SIJ-related disease may have been undocumented despite being present, which may limit the validity of our results to some extent. The impact of a number of parameters such as precise weight and height could not be investigated in depth, as this information could only be captured in a categorical fashion. Additionally, a cross-sectional study design is less suited to capture the evolution of such lesions than a longitudinal study design, as some of the contributing factors (e.g., weight) may change substantially over a person’s lifetime.

In conclusion, our results paint a complex picture of the development of degenerative lesions of the sacroiliac joints in a large asymptomatic study population. Anatomical SIJ variants have an impact on the development of such lesions and are very common in women while rare in men. Further analysis warranted to improve our understanding of the major difference in SIJ degeneration between men and women as well as to further elucidate the effects of altered biomechanics in individuals with atypical SIJ anatomy.

## Data Availability

The datasets generated during and/or analysed during the current study are available from the corresponding author on reasonable request.
